# The Pivotal ‘P’s of inflammatory bowel disease: Prediction and Prevention

**DOI:** 10.1002/ueg2.12519

**Published:** 2024-01-02

**Authors:** Dotan Yogev, Dan Turner

**Affiliations:** ^1^ Juliet Keidan Institute of Pediatric Gastroenterology and Nutrition, The Eisenber R&D Authority Shaare Zedek Medical Center Jerusalem Israel; ^2^ The Hebrew University of Jerusalem Jerusalem Israel

**Keywords:** epidemiology, IBD, prediction of IBD, prevention

Despite numerous advancements in the treatment of inflammatory bowel diseases (IBDs), many patients still face a debilitating disease course with complications, surgeries and frequent periods of active disease.^1^, ^2^ Moreover, while the incidence of IBD has started to plateau in many Western countries, the prevalence continues to rise in all parts of the world, and is expected to surpass 1% in the near future.^3^, ^4^ Once the prevalence in the developing world will reach that of the Western world, which is expected within the next 30 years,^4^ it is likely that the overall global resources required to treat IBD will fall short of those required for optimal treatment. For example, over 30 million IBD patients will be living in India and China alone based on the current Western incidence rates, which is at least three times higher than the number of IBD patients living in the entire world in 2022. This alarming projection calls for concerted action to focus not only on improving disease management but also on primary and secondary prevention strategies.

Two pillars lay at the foundation of disease prevention: identifying an at‐risk population (i.e., ability to predict disease) and applying an appropriate intervention. In addition to being effective, the latter needs to be practical, safe and feasible from the standpoint of an otherwise healthy individual. Possible preventive measures may include pharmaceutical management, microbiome manipulation, lifestyle changes (e.g., smoking reduction and physical activity), and dietary treatment.

First‐degree relatives of IBD patients may be candidates for preventive interventions, but biomarkers are needed to further select the most appropriate individuals for intervention. Studies have suggested several such biomarkers, from the fields of proteomics,^5^ serologics,^6^ and genetics, including fecal calprotectin.^7^ In the era of artificial intelligence, a different approach may facilitate interpretation of minor alterations in blood tests performed routinely for any indication in the preclinical phase. We and others have recently shown that minor alterations in blood tests occur years prior to IBD diagnosis.^8^, ^9^ Cohen et al. retrospectively analyzed routine blood tests of 5643 patients with IBD demonstrating significant changes occurring 2–5 years before diagnosis.^8^ In a different study, we included 7630 patients with Crohn's disease and showed that up to 24 routine blood tests showed significant differences versus non‐IBD matched controls up to 6 years pre‐diagnosis.^9^ These subtle changes, some of which were within the normal range, included intuitive tests such as white blood cells, platelets, albumin and C‐reactive protein but also tests that were seemingly not related to IBD such as cholesterol, creatinine, and bilirubin.

In this issue, Lerchova et al. address the issue of lifestyle interventions for disease prevention with an elegant retrospective analysis of prospectively enrolled cohorts, exploring whether screen‐time and physical activity are associated with risk of future IBD.^10^ Data were obtained from two Scandinavian birth cohorts assembled during 1997–2009, with the main variables collected at age 3 (*n* = 65,978) and 8 years (*n* = 38,506); 266 patients from the cohorts eventually developed IBD. Longer screen‐time at 8 years was associated with subsequent development of IBD (aHR = 1.51 [95%CI = 1.02–2.25]) but the level of physical activity was not. The prospectively collected data minimized recall bias but many possible unmeasured confounding factors could explain this association, including dietary habits associated with a sedentary lifestyle, rural versus urban residence, socioeconomic status and many more. Therefore, the conclusions of this study are limited to association and not causation. Still, the authors' work is important as it speaks toward the key role that lifestyle may play in disease prevention.

Two clinical trials are currently ongoing with the aim of preventing IBD, excitingly representing the first such trials in the history of gastroenterology. Our group is leading the Preventing IBD Onset in Individuals at Risk (PIONIR) trial (ClinicalTrial.GOV #NCT05211518), which is a crossover randomized controlled trial, including first‐degree relatives of patients with IBD participating in the Genetic Environment and Microbe (GEM) prospective cohort and having at least one other risk factor (e.g., elevated microbiome risk score, increased gut permeability, multiplex family and mildly elevated calprotectin despite normal or near normal panenteric capsule endoscopy). Participants are being randomized in a crossover design to the Tasty&Healthy dietary intervention or habitual diet and their risk biomarkers are being measured at 8 and 16 weeks. The Modulating Early Life Microbiome through Dietary Intervention in Pregnancy trial is exploring whether a dietary intervention during pregnancy of IBD mothers can change the microbiome of their newborns to mimic that of healthy infants, with the hopes that this will reduce the lifetime risk of developing IBD (ClinicalTrial.Gov # NCT03850600).

The road to accurate selection of individuals for primary and secondary prevention and identifying the most appropriate intervention for prevention is still long and challenging. However, it has real potential to be a game changer in our fight against IBD and the current study is another step toward that ambitious goal.

## CONFLICT OF INTEREST STATEMENT

The authors have no conflicts of interest to declare.

## Data Availability

This is an editorial not a research manuscript.
